# Descriptive epidemiology of Lassa fever in Nigeria, 2012-2017

**DOI:** 10.11604/pamj.2020.37.15.21160

**Published:** 2020-09-03

**Authors:** Onyebuchi Augustine Okoro, Eniola Bamgboye, Chioma Dan-Nwafor, Chukwuma Umeokonkwo, Elsie Ilori, Rimamdeyati Yashe, Muhammad Balogun, Patrick Nguku, Chikwe Ihekweazu

**Affiliations:** 1 Nigeria Field Epidemiology and Laboratory Training Program, Abuja, Nigeria,; 2 Department of Epidemiology and Medical Statistics, University of Ibadan, Ibadan, Nigeria,; 3 Nigeria Centre for Disease Control, Abuja, Nigeria,; 4 Department of Community Medicine, Alex Ekwueme Federal University Teaching Hospital Abakaliki, Ebonyi State, Nigeria,; 5 African Field Epidemiology Network, Nigeria Country Office, Abuja, Nigeria

**Keywords:** Lassa fever, Nigeria, outbreak

## Abstract

**Introduction:**

Lassa fever, an acute viral hemorrhagic zoonotic disease is endemic in some parts of Nigeria. The disease alert and outbreak threshold are known; however, there has been a shift from the previous seasonal transmission pattern to an all year-round transmission. We described data on Lassa fever and highlighted the magnitude of the disease over a six-year period.

**Methods:**

we conducted a secondary data analyses of Lassa fever specific surveillance data from the Integrated Disease Surveillance and Response (IDSR) records of all states in Nigeria over a six-year period (2012-2017).

**Results:**

a total of 5974 suspected cases were reported within the study period; of these, 759 (12.7%) were confirmed by laboratory diagnosis. Highest number of cases was recorded in 2012. Edo and Ondo states in the southern region of the country were mostly affected within the study period. The seasonal trend of Lassa fever cases showed peaks within January to March, except for year 2015.

**Conclusion:**

there was a high burden of Lassa fever in Nigeria especially in the southern part. Lassa fever transmission occurs all year-round with peaks in January and March. There is need to develop preparedness plans and define thresholds for Lassa fever epidemic.

## Introduction

Lassa fever is a viral haemorrhagic fever caused by a single stranded RNA virus belonging to the Arenaviridae family, it is a zoonotic disease whose reservoir is the multimammate rat of the genus *Mastomys* [1]. Humans are infected by exposure to food or household items contaminated with excreta or urine of infected rodents, processing of infected rats for consumption, airborne through the inhalation of tiny particles in the air contaminated with infected rodent excretions or reuse of infected needles [1-3]. Person-to-person transmission also occur especially among healthcare workers through direct contact with body fluids of infected persons, often due to a lack of appropriate infection, prevention and control (IPC) measures whilst receiving care [4, 5]. Lassa fever was first discovered in 1969 in Nigeria following the death of two missionary nurses in Lassa town, Borno State [6]. The disease is endemic in West Africa countries of Sierra-Leone, Liberia, Guinea and Nigeria where about 300,000 to 400,000 cases occur annually with approximately 5,000 deaths [7]. Cases have also been reported in Central African Republic, Democratic Republic of the Congo, Mali and Senegal [5, 7-10]. Lassa fever cases are difficult to differentiate from other febrile illness and if not well managed could result to high fatality rates [11]. In Nigeria, Lassa fever is one of the seven epidemic prone notifiable diseases reportable under the Integrated Disease Surveillance System (IDSR), a suspected case is considered an alert threshold and one confirmed case an epidemic threshold. Sporadic outbreaks occur annually, and have been reported in up to one-third of states in the country [1, 2, 7, 12, 13]. There is however paucity of publications on the magnitude of the disease in the country. This could affect early preparedness and resource allocation which helps in the control of the disease. This study was therefore conducted to epidemiologically describe Lassa fever infections highlighting the magnitude of the disease in Nigeria over a six-year period.

## Methods

**Study setting:** Nigeria has 36 states and a Federal Capital Territory (FCT) with 774 Local Government Areas (LGAs) and is majorly categorized into six geopolitical regions (South East, South South, South West, North Central, North West and North East). The country has two distinct seasons which includes rainy season starting between March and May and ending between September/November, depending on the regions. The dry season starts in October/December, and ends in April and may extend to May/June in other areas. Lassa fever surveillance in Nigeria is through the IDSR platform. Information flows from the health facilities, through the ward focal persons to the Local Government Areas (LGA) Disease Surveillance and Notification Officers (DSNOs), to the State DSNOs, and then to the Federal Ministry of Health (Nigeria Centre for Disease Control) through the State Epidemiologist. Feedback goes through the opposite direction. All states in Nigeria including FCT report through the IDSR.

**Study design and population:** secondary data analyses of Lassa fever specific IDSR records in Nigeria from 2012-2017 was conducted. IDSR weekly epidemiological data line list for the years under review was obtained from the surveillance and epidemiology department, Nigeria Center for Disease Control (NCDC). The variables reported were state of residence, and Lassa fever classification based on laboratory diagnosis (suspected, confirmed).

**IDSR Lassa fever case definitions:** a suspected case was defined as any person with an illness of gradual onset with one or more of the following: malaise, fever, headache, sore throat, cough, nausea, vomiting, diarrhoea, myalgia (muscle pain), central chest pain or retrosternal pain, hearing loss and either a history of contact with excreta or urine of rodents or history of contact with a probable or confirmed Lassa fever case within a period of 21 days of onset of symptoms or any person with inexplicable bleeding. A probable case was defined as any suspected case as defined above but who died or absconded without collection of specimens for laboratory testing and a confirmed case as any suspected case with laboratory confirmation (positive IgM antibody, PCR or virus isolation). The definitions were according to the NCDC guideline for management of Lassa fever.

**Data management:** we sorted, extracted and cleaned relevant data on number of cases, location and laboratory results (positive or negative). We examined the data for completeness. We calculated frequencies and proportions using Microsoft Excel 2016, maps were generated with Quantum Geographic Information System (QGIS) software.

**Ethical considerations:** approval was received from the surveillance and epidemiology department of Nigeria Centre for Disease Control.

## Results

**Figure 1:**
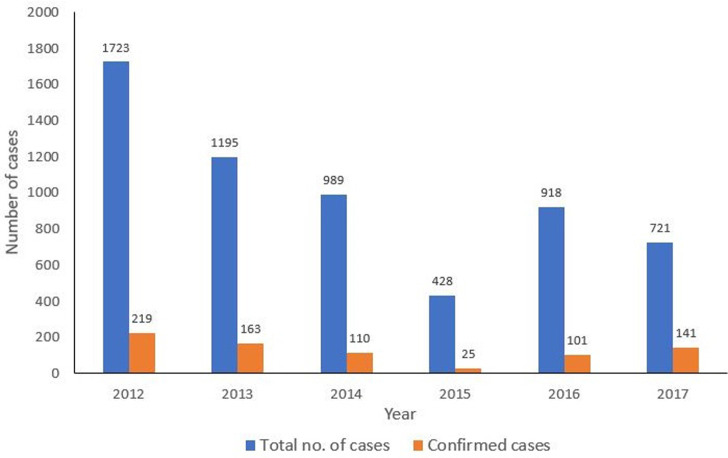
annual distribution of Lassa fever cases in Nigeria, 2012-2017

**Figure 2:**
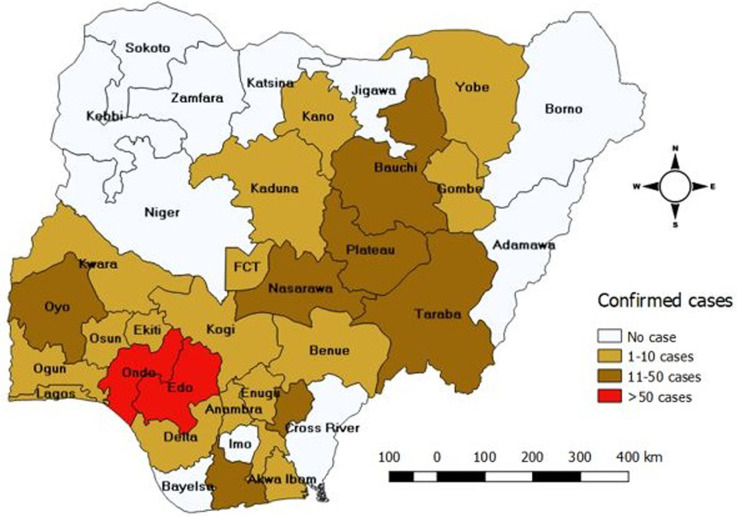
distribution of confirmed Lassa fever cases in Nigeria, 2012-2017

**Figure 3:**
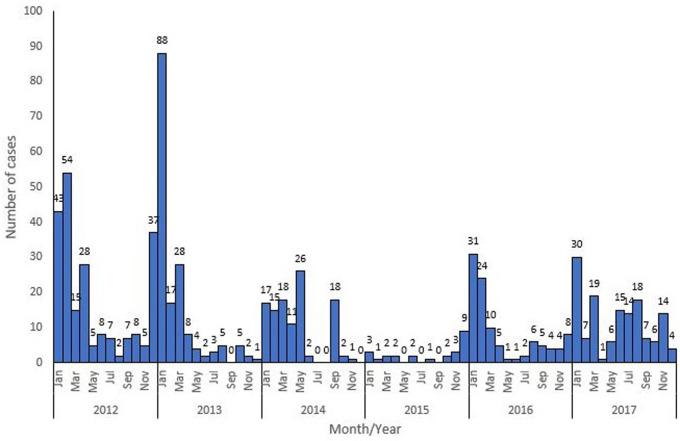
monthly trend of confirmed Lassa fever cases in Nigeria, 2012-2017

A total of 5,974 cases were reported between January 2012 to December 2017, of these number, 759 (12.7%) were confirmed cases. Highest number of cases 1723 (28.8%) were reported in 2012, followed by 2013 with 1195 (20.0%). The least number of cases 428 (7.2%) were reported in 2015 (Figure 1). Of all the confirmed cases, states in the southern region were more affected with all states in the region except Bayelsa, Imo and Cross River reporting at least one confirmed case. Edo and Ondo states reported the highest number of cases (> 50 confirmed cases). More states in the Northern region did not have any confirmed case within the study period (Figure 2). The trend of Lassa fever cases showed a pattern but wasn´t consistent throughout the six-year period. Cases were seen to peak between January to March each year except in 2015 and 2016 where cases peaked in May and December respectively. There was also an almost all year-round occurrence of cases within the six-year period (Figure 3).

## Discussion

Our study shows a high magnitude of Lassa fever disease in Nigeria, especially in the southern part of the country, the high number of confirmed cases reported within the six-year study period raises concerns considering the high morbidity and mortality associated with Lassa fever infections particularly in the absence of early diagnosis and proper management. Our findings corroborates earlier reports of endemicity of Lassa fever in West Africa region including Nigeria [7, 14]. The high number of cases from Edo and Ondo could either be due to the presence of the reservoir in the states [15], or cultural and environmental practices; like drying of foodstuff outside, in addition to the presence of a major treatment centre in Edo state where most Lassa fever cases from the country are managed [16]. The staggered pattern in peak occurrence of confirmed cases and an almost all year round occurrence of cases within the six-year study period suggests that the earlier reports of seasonal variation in occurrence of cases [2, 11] shouldn´t be solely relied on for preparedness activities. It also corroborates documented reports that Lassa fever outbreaks could occur anytime of the year [7, 12]. There is therefore need to maintain a high index of suspicion amongst health care workers [17], develop and maintain a country outbreak preparedness plan and intensify risk communication activities. Geographically, our study shows a spread of cases in 26 states of the country and across all the geo-political regions. This differs from the reports of other studies where cases were reported in fewer states than we found [14, 18]. The spread of cases across states including states that share borders with other countries suggests a risk for inter-border transmission and possess a threat to global health security. This calls for cross board collaboration in management of Lassa fever epidemic and need for information sharing between countries to minimize the risk of international spread. Edo State consistently had the highest number of cases within the six-year study period, this might be due to the presence of a major and foremost treatment centre in the state which could make patients seek treatment in the centre without notifying their originating states as recommended in IDSR system. It might also be due to the presence of the reservoirs [15], or cultural practices with regards to food processing and preservation. The high proportion of cases in the southern region when compared with the northern region calls for further research on the ecology of Lassa virus, particularly when we consider that the first reported case of Lassa fever in the country was from the northern region [6] and genetic sequencing of positive 2018 Lassa fever samples elucidated no changes in the viral genome or transmission patterns [19].

**Limitations:** this study was burdened with the several limitations of secondary data analysis including absence of some variables that could have helped perform a complete descriptive analysis. Also, the data was not specific on case classifications, there were no records of probable cases and diagnostic outcome for all reported cases.

## Conclusion

Our study has shown an increasing magnitude of Lassa fever in Nigeria with endemicity in most southern states of the country, there is therefore need to redefine the disease threshold level across states, develop and institute preparedness plans at national and subnational levels and establish more diagnostic and treatment facilities with competent workforce across the six geo-regions of the country.

### What is known about this topic

- Lassa fever is endemic in West Africa;
- Lassa fever infections are associated with high morbidity and mortality.

### What this study adds

- This study has shown an increased magnitude of Lassa fever in Nigeria;
- The southern region of the country has repeatedly been most affected;
- The need to review the disease threshold based on the high occurrence of the disease.
